# Supplementary material for: From sequence to enzyme mechanism using multi-label machine learning
Source: BMC Bioinformatics. 2014 May 19;15:150. doi: 10.1186/1471-2105-15-150 (PMC4229970; doi:10.1186/1471-2105-15-150)
Supplement: Additional file 2 — Java code of ml2db. Additional file ml2db_code.tar.gz contains the Java source code to run the multi-label machine learning experiments and save the results to database. The code’s Javadoc is included. [file 1471-2105-15-150-S2.zip › additional file 2/ml2db/ml2db/doc/main/ml2db/weka/ImportArff.html]

ImportArff


JavaScript is disabled on your browser.


- Overview
- Package
- Class
- Use
- Tree
- Deprecated
- Index
- Help

- Prev Class
- Next Class

- Frames
- No Frames

- All Classes

- Summary:
- Nested |
- Field |
- Constr |
- Method

- Detail:
- Field |
- Constr |
- Method


main.ml2db.weka

## Class ImportArff

- java.lang.Object
- - main.ml2db.weka.ImportArff

- Direct Known Subclasses:
  :   ImportMulanArff

  ---

    

  ```
  public class ImportArff
  extends java.lang.Object
  ```

  Class to import an existing Weka ARFF file to a record in the arff database table.
  The resulting record identifier (id) can then be used to run evaluation, prediction etc. on the file.
  Input: a valid ARFF file path and (optional) a description for the data
  Output: an integer identifier of this file database record

  Version:
  :   5 Mar 2014

  Author:
  :   Luna De Ferrari ldf@st-andrews.ac.uk

- - ### Constructor Summary

    Constructors

    Constructor and Description

    `ImportArff(java.lang.String arffFilePath,
    java.lang.String dbPropsFilePath,
    java.lang.String description)`
  - ### Method Summary

    Methods

    | Modifier and Type | Method and Description |
    | --- | --- |
    | `java.lang.String` | `getArffFilePath()` |
    | `uk.ac.standrews.utils.main.database.DbManager` | `getDbConn()` |
    | `java.lang.String` | `getDescription()` |
    | `static void` | `main(java.lang.String[] args)` IMPORTANT: Please note that the arff to be imported should have, before each instance line, a line starting with % containing a unique instance identifier. |
    | `int` | `recordArff()` |

    - ### Methods inherited from class java.lang.Object

      `equals, getClass, hashCode, notify, notifyAll, toString, wait, wait, wait`

- - ### Constructor Detail


    - #### ImportArff

      ```
      public ImportArff(java.lang.String arffFilePath,
                java.lang.String dbPropsFilePath,
                java.lang.String description)
      ```

      Parameters:
      :   `arffFilePath` -
      :   `propertiesFilePath` -
      :   `description` - the database to save the arff file record to
  - ### Method Detail


    - #### getArffFilePath

      ```
      public java.lang.String getArffFilePath()
      ```


    - #### getDescription

      ```
      public java.lang.String getDescription()
      ```


    - #### getDbConn

      ```
      public uk.ac.standrews.utils.main.database.DbManager getDbConn()
      ```


    - #### recordArff

      ```
      public int recordArff()
      ```

      Returns:


    - #### main

      ```
      public static void main(java.lang.String[] args)
      ```

      IMPORTANT: Please note that the arff to be imported should have, before each instance line,
      a line starting with % containing a unique instance identifier.
      Example for a sparse arff:


- Overview
- Package
- Class
- Use
- Tree
- Deprecated
- Index
- Help

- Prev Class
- Next Class

- Frames
- No Frames

- All Classes

- Summary:
- Nested |
- Field |
- Constr |
- Method

- Detail:
- Field |
- Constr |
- Method
